# Cryptogenic embolic stroke and cancer

**DOI:** 10.3389/fneur.2025.1537779

**Published:** 2025-06-13

**Authors:** Joaquin Carneado-Ruiz

**Affiliations:** ^1^Neurology Department, Medicine Universidad Autonoma de Madrid, Madrid, Spain; ^2^Stroke Unit, Neurosonology Laboratory, Hospital Universitario Puerta de Hierro Majadahonda, Majadahonda, Spain

**Keywords:** cryptogenic embolic stroke, Cancer, MRI, d-dimer, review

## Abstract

Oncologic and cerebrovascular diseases are among the diseases with the highest incidence rate and are leading causes of disability and mortality. The relationship between cancer and cerebrovascular disease has been studied for decades, yet it remains a challenge. Stroke, in relation to oncologic diseases, has particularities in its diagnosis and treatment. Cancer is an established risk factor for ischemic stroke. The highest risk of stroke occurs within the first 6 months after a cancer diagnosis and in patients with metastases. Between 2 and 10% of patients initially diagnosed with cryptogenic stroke are subsequently diagnosed with cancer within 1 year. The mechanism underlying cryptogenic ischemic stroke associated with oncologic disease is acquired hypercoagulability, which is the most frequent mechanism underlying stroke in patients with cancer. Sometimes, cancer presents itself as non-bacterial thrombotic endocarditis (NBTE) with cerebral infarction. Strokes are usually more severe, and their clinical presentation can be focal or multifocal. D-dimer levels are significantly elevated in patients with cancer-associated stroke. Magnetic resonance imaging (MRI) usually shows embolic lesions across several arterial territories, including both carotid territories and the vertebrobasilar territory. Patients with cancer-associated stroke face a higher risk of recurrence, recurrent thromboembolism, early neurological deterioration, and mortality. Patients with both stroke and cancer should be considered for thrombolysis (recombinant tissue plasminogen activator (rTPA) or tenecteplase) and endovascular treatment. Low-molecular-weight heparin is usually used empirically when a hypercoagulable state is suspected, and few studies have supported the use of direct oral anticoagulants as an option with similar efficacy. The objective of this review was to synthesize all relevant information available to date on neoplasia as a cause of cryptogenic embolic stroke and to provide useful insights for everyday clinical practice.

## 1 Introduction

Oncologic and cerebrovascular diseases have the highest incidence rate and are leading causes of disability and mortality. Approximately 40% of the population faces a lifetime risk of developing cancer (1, 2). The lifetime risk of stroke from the age of 25 years is 25% (3).

The connection between cancer and cerebrovascular disease has been a subject of study for decades. The first large series of autopsy studies in 1985 showed that 14.6% of patients with cancer had cerebrovascular disease and half of the cases were symptomatic. The main neurological complication associated with brain metastasis is cerebrovascular disease (ischemic and hemorrhagic) (4).

In the general population, most strokes are ischemic, and one-third of the cases remain cryptogenic with no recognized etiology (5). Cryptogenic ischemic strokes can be divided into subgroups, some of which have particularities in their diagnosis and treatment, such as stroke related to oncologic disease. This type of stroke occurs in patients with active cancer; less frequently, patients will have occult neoplasia, which, if diagnosed, will offer an enormous possibility for treatment and improvement in prognosis (6).

In recent years, a substantial body of scientific evidence has been generated that allows us to identify the specific characteristics of this association between stroke and cancer.

The objectives of this review were as follows: to synthesize all relevant information available to date on cancer as a cause of cryptogenic embolic stroke (CES-ONC) and to provide useful insights for everyday clinical practice that can help improve the management of patients with cancer and ischemic stroke.

## 2 Epidemiology

Of the population of patients with cancer, 15% have cerebrovascular disease (7, 8), and the frequencies of ischemic and hemorrhagic strokes are similar (8). Among patients with stroke, 10% have a history of cancer (7), and the prevalence of cancer in this group is higher than in the general population (9, 10). In the cryptogenic stroke subgroup, 10% of patients also have a history of cancer (7).

However, attributing the etiology of stroke to cancer presents two challenges:

1. Some of these associations might be coincidental.
2. A subgroup of patients with stroke may have occult neoplasms at the time of diagnosis, which could be the underlying cause.

The co-prevalence of stroke and cancer is expected to rise owing to an increase in the survival rate of patients with cancer. Registries of patients with oncologic diseases show an increase in survival in patients with lung, breast, and prostate cancers, the three types of cancer with the highest incidence rate (11).

Cancer is an established risk factor for ischemic stroke. Multiple studies, including prospective studies, have demonstrated an increased risk of ischemic stroke and other arterial thromboembolic events in patients with incident oncologic disease compared to controls (5, 12). The highest risk of stroke occurs within the first 6 months after a cancer diagnosis and in patients with metastases (12). The risk of stroke varies according to the type of stroke and is higher in cancers associated with pulmonary thromboembolism, particularly lung and pancreatic cancers (13, 14).

In patients with both stroke and cancer, 10% present with venous thromboembolism (10, 15, 16). In a population of patients with venous thrombosis of unknown etiology, the prospective randomized screening study Screening for Occult Malignancy in Patients With Idiopathic Venous Thromboembolism (SOME) found that only 3.9% of the patients were diagnosed with cancer within the following year.

Regarding the issue of occult neoplasia at the time of stroke diagnosis, between 2 and 10% of patients with cryptogenic stroke are diagnosed with cancer within a year of diagnosis (17–19).

The most frequently occurring neoplasms in patients with stroke are urogenital, breast, and gastrointestinal neoplasms. A higher incidence of stroke has been reported in patients diagnosed with lung, pancreatic, colorectal, breast, and prostate cancers (20).

## 3 Etiopathogenia

The type of tumor most commonly associated with embolic stroke of undetermined etiology is adenocarcinoma. However, all types of cancer, whether solid or hematologic, and at any stage, are associated with an increased risk of stroke.

The potential mechanisms through which cancer can cause stroke include cancer-related acquired hypercoagulability, direct invasion or compression of arteries, infection, and side effects of radiotherapy or chemotherapy.

The mechanism underlying CES-ONC is acquired hypercoagulability, which is the most frequently observed mechanism of stroke in patients with cancer. This state of hypercoagulability explains the high frequency of thrombotic events (both venous and arterial) in patients with cancer (21).

In the case of other mechanisms, there is a well-defined and identifiable etiology, and one could not speak of stroke without a known cause.

The pathobiology underlying a hypercoagulable state is complex and varies according to the type of cancer, its histology, and multiple interconnected factors. An increase in procoagulant factors, including tissue factors, mediated by both cancer cells and the systemic inflammatory response, is observed.

Stroke of unknown etiology in patients with a history of oncologic disease has a different molecular profile and peripheral blood gene expression (mRNA) compared to isolated cancer or isolated stroke (22, 23). There is an increase in the number of extracellular vesicles derived from cancer cells and platelets. These vesicles trigger a hypercoagulable state (24). In the OASIS-Cancer study, cancer cell-derived extracellular vesicles were found to correlate with D-dimer levels, which triggered hypercoagulability independently of tissue factor-dependent pathways. In patients with lung cancer, the subtype associated with vesicle elevation is adenocarcinoma (25).

Another factor is neutrophil extracellular trap formation, which is a part of the innate immune response and promotes platelet and coagulation factor activation. Patients with CES-ONC have increased levels of neutrophil extracellular trap formation, which are associated with thrombin–antithrombin complex, a marker of coagulation, and P-selectin, a marker of platelet activity (26).

In the OASIS-Cancer study, circulating plasma DNA and nucleosome levels, markers of neutrophil extracellular trap formation, were associated with high levels of D-dimer and were higher in patients with CES-ONC than in controls (22).

Another relevant factor is the presence of platelets with high activity and an increased tendency to aggregate. Extracellular vesicles derived from platelets and related to tissue factors are elevated in all types of stroke, regardless of whether the etiopathogenetic mechanism is associated with neoplasia.

### 3.1 The etiopathogenetic mechanisms through which cancer causes embolic stroke of undetermined etiology are as follows (20)

#### 3.1.1 Related to hypercoagulability

##### 3.1.1.1 Sterile vegetation on cardiac valves in the context of acquired hypercoagulability

In this case, cancer usually metastasizes and cerebral infarction is a late-stage complication. Occasionally, non-bacterial thrombotic endocarditis (NBTE) with cerebral infarction can be an initial manifestation of cancer (27). In a population of cancer patients, NBTE occurs in 9.3–19% of cases, while in patients diagnosed with NBTE, cancer can be found in 59% (28–31). Patients with emboli associated with NBTE have high platelet counts and low erythrocyte fractions (32).

##### 3.1.1.2 Through disseminated vascular coagulation

In this case, the condition meets the criteria for disseminated intravascular coagulation associated with thrombopenia and hypofibrinogenemia. Strictly speaking, it cannot be considered a stroke of undetermined etiology.

##### 3.1.1.3 Paradoxical embolism through a patent foramen ovale

In this case, 25% of the population has a patent foramen ovale as a remnant of fetal circulation. This is primarily due to hypercoagulability and the high risk of venous thrombosis.

#### 3.1.2 Other mechanisms not related to hypercoagulability or cryptogenic etiology

Cancer can cause stroke through other well-defined etiologies that are infrequent and, therefore, cannot be considered cryptogenic.

##### 3.1.2.1 Atherothrombosis may be associated with cancer risk factors, including obesity, carbohydrate intolerance, and smoking

Radiotherapy can cause arterial injury and destabilize atheroma plaques within months, especially when combined with the pro-inflammatory effects of cancer. Atheroma plaques in the aortic arch must also be considered because thoracic radiation is common in breast cancer or lymphoma. It can also damage the coronary arteries, cardiac valves, myocardium, and pericardium, leading to embolic stroke (33).

##### 3.1.2.2 Antineoplastic treatments

Generally, the risk of stroke associated with chemotherapy is low. However, this risk is higher with certain treatments, including methotrexate (MTX), 5-fluorouracil, cisplatin, and L-asparaginase (34, 35). Anthracycline chemotherapy can also lead to chronic cardiomyopathy. All agents with anti-estrogenic effects can increase the risk of stroke (36, 37). Immunotherapy, which is used in modern treatment regimens, can lead to vasculitis and myocarditis (38, 39).

Tumor embolism may be a mechanism of embolic stroke. This mechanism occurs when a tumor invades the pulmonary vein or cardiac cavity.

Embolic strokes can occur during tumor surgery due to tumor embolism, direct injury to the arteries, or cardiac arrhythmias associated with surgical intervention.

## 4 Clinical characteristics

The clinical characteristics of patients with CES-ONC can be defined as follows (20):

1. The traditional risk factors shared by cancer and stroke are obesity, carbohydrate intolerance, and smoking. In general, patients with an association between cancer and stroke have fewer traditional vascular risk factors than patients who have a stroke without cancer.
2. The oncologic antecedent most closely related to embolic stroke of undetermined etiology is adenocarcinoma, although all types of cancer, whether solid or hematologic, at any stage, are associated with an increased risk of stroke.
3. Strokes are usually more severe; therefore, we should consider the degree of previous disability due to oncologic disease as a possible confounding factor.
4. Its clinical presentation can be focal or multifocal. In 30–70% of cases, neuroimaging shows emboli lesions in several arterial territories, including both carotid territories and the vertebrobasilar territory (20, 25).
5. They face a higher risk of recurrence, recurrent thromboembolism, early neurological deterioration, and mortality.

## 5 Diagnostic considerations

In patients with a history of cancer and stroke of undetermined etiology, the diagnostic challenge lies in detecting and stratifying the importance of the relationship, ultimately establishing an etiopathogenetic link between ischemic stroke and acquired hypercoagulability associated with cancer. This condition not only increases the risk of venous thrombosis but also of arterial thrombosis (14).

Another diagnostic challenge in this context is that one of the causes of stroke of unknown etiology is the presence of occult neoplasia, which occurs in 2.8% of patients. However, the optimal screening strategy remains unclear. Moreover, biomarkers with adequate sensitivity and specificity to aid in early diagnosis are not yet available. This constitutes a relevant current diagnostic problem, without an adequate solution (6).

Approximately 50% of strokes in patients with oncologic disease are of undetermined etiology, a higher percentage compared to patients without cancer (8–11, 40, 41).

A high index of suspicion for acquired hypercoagulability associated with cancer should be maintained in patients with a history of oncologic disease and stroke of undetermined etiology (14). The condition is characterized by hypercoagulability rather than consumption coagulopathy.

In the case of venous thrombosis of unknown etiology, the prospective randomized screening study, SOME, found that only 3.9% of patients with venous thrombosis of unknown etiology were diagnosed with cancer within the following year. There were no diagnostic differences between the group of patients assigned to the computed tomography (CT) screening and the group of patients assigned to the basic evaluation, which included analysis, chest X-ray, and age- and sex-appropriate screening for breast, cervical, and prostate tumors.

Further studies with similar design are needed to be able to advise on the type of screening that is most appropriate for patients with cryptogenic embolic stroke and suspected occult neoplasia (12). Occult neoplasia is identified in 2–10% of embolic ischemic stroke cases; this diagnosis is made within a year after the stroke (17–19).

### 5.1 Biomarkers (42, 43)

D-dimer levels are significantly increased in patients with cancer-associated stroke of undetermined etiology compared to patients with stroke of conventional etiology (6.15 [standard deviation {SD}: 8.5] vs. 1.39 [SD: 1.9] in units of μg/mL) (10, 15). Most patients with CES have increased levels of inflammatory factors and D-dimer, although this profile occurs in cancer in general and in other stroke etiopathogenetic mechanisms (e.g., cardioembolic).

Other potential biomarkers suggesting neoplasia as the etiology of stroke are C-reactive protein (CRP) and fibrinogen. CRP levels of >20 mg/L have a sensitivity of 75% and specificity of 96%, whereas fibrinogen levels of >600 mg/dl have a sensitivity of 67% and specificity of 91% for ischemic stroke associated with neoplasia. Data from patients with lung cancer indicate that D-dimer, CA125, CA199, and CRP are biomarkers associated with this type of neoplasia.

Other possible factors that could be biomarkers (Table 1):

**Table 1:** Possible factors that could be biomarkers.

Factors
Tissue factors.
Hematogenous extracellular vesicles derived from cancer cells and platelets (24).
MicroRNAs contained in cancer cell-derived extracellular vesicles (82).
Neutrophil extracellular trap formation (NETosis) (26).
Circulating plasma DNA and nucleosome levels, purported markers of NETosis (26).
Abnormal platelet activity with increased aggregation.
Increased von Willebrand factor levels.
Several endothelial markers (thrombomodulin, soluble intercellular adhesion molecule-1, vascular cell adhesion molecule-1).
Tumor expression of fibrinolysis inhibitors and inflammatory cytokines.
Factors related to adenocarcinomas: production of mucin.
Study of the thrombus extracted using the endovascular treatment technique (44, 45).

Related to adenocarcinomas: The production of mucin, a high molecular weight molecule that is glycosylated and secreted normally by endothelial cells, causes hypercoagulability. Adenocarcinomas: The pancreas, colon, breast, lung, prostate, and ovarian systems secrete this molecule into the bloodstream.

Analysis of the thrombus extracted using endovascular treatment can provide information about the etiological subtype. In a histopathological study, patients with active cancer had higher platelet counts and lower erythrocyte fractions (“white clots”) than those with inactive cancer and no cancer (44). Immunohistochemical assessments may offer more precise information for the diagnosis of cancer-associated stroke, and this type of analysis has achieved high diagnostic accuracy in identifying cancer-associated stroke, with areas under the curve ranging from 0.946 to 0.986. It has been demonstrated that it could predict occult cancer with probabilities ranging from 88.5 to 99.2% (45).

### 5.2 Neuroimaging markers: magnetic resonance imaging (MRI) and diffusion-weighted imaging (DWI)

In 30–70% of CES-ONC cases, embolic lesions are detected in several arterial territories, with the three-territory sign on MRI DWI commonly observed— characterized by multiple emboli in both carotid territories and the vertebrobasilar territory (46–51).

#### 5.2.1 Biomarkers associated with MRI and DWI

Elevated CRP and D-dimer levels are associated with the neuroimaging patterns of multiple lesions (46). D-dimer levels greater than 0.55 mg/L, along with the presence of cerebral infarcts in multiple locations, have a specificity and positive predictive value of 99.7 and 92.9%, respectively, for cancer-related CES-ONC. When neuroimaging findings were not included, D-dimer levels of ≥5.5 mg/L had a high specificity of 99.6%, although the sensitivity level dropped considerably to 31.9% (47). We suspect occult neoplasia in patients with CES when abnormally elevated D-dimer levels or a combination of elevated D-dimer and MRI findings are present (47, 52).

### 5.3 Practical conclusion for diagnosis

Increased D-dimer levels are useful as a biomarker for CES-ONC. Other possible candidates of biomarkers include CRP and fibrinogen levels; however, these require further confirmatory studies.

More specific tumor markers have not proven to be useful as CES-ONC biomarkers (43).

The best results in terms of specificity and positive predictive value were obtained by combining elevated D-dimer levels and the presence of cerebral infarcts in multiple locations.

An optimal diagnostic study protocol for ischemic stroke should be followed, and evidence of cryptogenic embolic cerebral infarction (etiology not clarified) may be obtained after a comprehensive evaluation.

The study and treatment of patients should be conducted using a protocol that includes a detailed clinical history collected in a semi-structured manner, a detailed neurological examination, and a standardized clinical evaluation. We recommend assessing the severity of the neurological deficit using the National Institutes of Health Stroke Scale and the Rankin Scale at admission and discharge or at 7 days and 90 days. In the first diagnostic approach, we recommend describing each of the etiological phenotypes of cerebral infarction, classified according to the ASCOD criteria (53, 54).

Routine analysis should include a complete blood count, urea, creatinine, total cholesterol, low-density lipoprotein cholesterol, high-density lipoprotein cholesterol, triglyceride subfractions, glucose, electrolytes, ultrasensitive CRP, and liver enzymes. A 12-lead electrocardiogram should be performed and repeated periodically if there is suspicion of arrhythmia. In addition, postero-anterior chest radiography, cranial CT, cranial MRI, and transthoracic echocardiogram should be conducted. Cardiac monitoring with automatic 24-h rhythm detection or a 24-h Holter electrocardiogram is also recommended. Imaging evaluation of the extracranial and intracranial arteries should include cerebral arteriography, MRI angiography, CT angiography, or duplex imaging of the supra-aortic and transcranial trunks. Angio-CT should also be conducted as an imaging test to assess the proximal aortic arch. A thrombophilia, immunological, and serology study (syphilis, Lyme disease, and human immunodeficiency virus) should be conducted if deemed necessary by the neurologists in charge of the patient. Special thrombophilia studies should be conducted only in patients with a personal or family history of disease or signs of unusual thrombosis.

There must be evidence that the etiopathogenetic mechanism of cerebral infarction is embolic in nature:

Lacunar infarction must be ruled out on control brain MRI (including DWI diffusion sequence) performed between day 2 and day 5.

Lacunar infarction is defined as a subcortical cerebral infarct with a diameter of less than 1.5 cm (≤2.0 cm on skull MRI using DWI sequences) within the territory of the perforating arteries.

A specific diagnostic study in a patient with suspected CES-ONC must added to the previous one:

A standard investigation, such as plasma D-dimer analysis, is a diagnostic and prognostic marker in these patients (55). A reduction in D-dimer levels after the initiation of antithrombotic therapy is associated with a lower risk of recurrence (56).

If the patient’s conditions are favorable, transesophageal echocardiography should be performed after transthoracic echocardiography as transesophageal echocardiography is useful for identifying certain cardioembolic mechanisms, including NBTE and aortic atheroma.

If a patent foramen ovale with a significant right-to-left shunt is identified, the following evaluation should be conducted: bilateral lower extremity venous ultrasound, upper extremity venous ultrasound if a central venous catheter is present, and CT of the chest to evaluate venous thromboembolism. Pelvic magnetic resonance venography may be useful (57). If venous thrombosis with paradoxical embolism is diagnosed, long-term anticoagulation therapy is recommended. In patients with stroke and cancer, venous thromboembolism occurs in approximately 10% of cases, and the majority of these patients exhibit elevated D-dimer levels. The diagnosis of venous thrombosis in this patient type is relevant for diagnosis, treatment, and prognosis (58).

Finally, as discussed previously, histopathological analysis with immunohistochemical assessments of the thrombus extracted using endovascular treatment can be useful for providing information on the etiological subtype (45).

## 6 Treatment

For CES-ONC, we must continue to monitor the patient’s vascular risk factors—smoking, high blood pressure, diabetes mellitus, hyperlipidemia, atrial fibrillation, and carotid stenosis.

It is recommended to not overlook possible coincidental etiologies that are susceptible to treatments other than those targeting hypercoagulability; recent data suggest that treatment with statins in vasculopathy due to radiotherapy may reduce the risk of stroke.

In relation to chemotherapy, treatments with an anti-androgen effect, such as most treatments for breast cancer, increase the risk of ischemic stroke.

### 6.1 Treating acquired hypercoagulable state in CES-ONC

There is uncertainty about the best way to treat an acquired hypercoagulable state, particularly regarding the most appropriate choice of antithrombotic agent.

Low-molecular-weight heparin is commonly used empirically when a hypercoagulable state is suspected, but the benefit is unclear, especially in patients with a high bleeding tendency.

A situation specific to these patients is the need for anticoagulation to prevent atrial fibrillation cardioembolism, extrapolated from the subgroup analysis of large randomized clinical trials on anticoagulation, recommending the use of direct-acting anticoagulants (DOACs) instead of antivitamin K.

Several small studies have compared different antithrombotic treatments; however, randomized trials are required.

The TEACH, a pilot trial, compared enoxaparin and aspirin in 20 patients with cancer and found no difference in the recurrence of thromboembolic events or survival rates. There was a problem of enrollment failure in the TEACH owing to patient reluctance to receive injections, and 40% of patients who were randomized to enoxaparin switched to aspirin because of discomfort with injections. This highlights a clear preference for the oral route in this patient group (58). The results of the Trial of Apixaban Versus Aspirin in Cancer Patients With Cryptogenic Ischemic Stroke (TEACH2) are still pending.

The Edoxaban for the Treatment of Coagulopathy in Patients With Active Cancer and Acute Ischemic Stroke (ENCHASE) pilot study (59) found that edoxaban and enoxaparin were comparable with respect to biomarkers of hypercoagulability and cerebral thromboembolism. Larger trials are warranted to compare the effects of edoxaban and enoxaparin on recurrent stroke and major bleeding events in patients with cancer-related ESUS.

A subanalysis of the NAVIGATE study showed that patients with embolic stroke of unknown etiology and a history of cancer experienced similar rates of ischemic stroke recurrence and mortality when treated with aspirin and rivaroxaban, which offers a better safety profile than rivaroxaban in terms of major bleeding (60).

The American Society of Clinical Oncology supports the use of DOACs for the treatment of cancer-associated venous thromboembolism, but it is not the same entity as stroke, and this guideline cautions that there are limited data on the risks and benefits of anticoagulation beyond 6 months.

Several randomized trials have shown that oral factor Xa inhibitors are comparable to sc low-molecular-weight heparin in terms of efficacy and safety for the prevention of venous thromboembolism and major bleeding in patients with cancer, making them a compelling option for the treatment of CES-ONC (61–63). Low-molecular-weight heparin agents are commonly used empirically when a hypercoagulable state is suspected, but the benefit is unclear, especially in patients with a high bleeding tendency. The studies we have conducted so far suggest that oral factor Xa inhibitors are comparable to sc low-molecular-weight heparin in terms of safety and efficacy in patients with cancer and a hypercoagulable state.

### 6.2 Treatment during the acute phase of stroke

#### 6.2.1 Treatment with intravenous thrombolysis

Cancer should not be considered a contraindication in itself for thrombolysis with recombinant tissue plasminogen activator (rTPA), as there is no evidence to suggest that the risk of complications is higher in cancer patients with this treatment.

However, there are no data on the results of tenecteplase in this patient type.

#### 6.2.2 Endovascular treatment

The thrombus formed due to the state of hypercoagulability secondary to cancer has specific characteristics because of its nature, being rich in platelets and poor in erythrocytes, which makes its extraction difficult. Therefore, in CES-ONC, thrombi retrieved during endovascular procedures tend to fragment easily.

Thromboembolic phenomena occur under conditions of high flow and hypercoagulability (64).

In the SECRET study, in which a group of patients with CES-ONC was compared to another group of patients with conventional stroke (without cancer or with inactive cancer), there was no significant difference between the groups in terms of the National Institutes of Health Stroke Scale score 24 h after treatment (median change in the score of 2.5 in the active cancer group vs. 3 in both the no cancer and non-active cancer groups, *p* = 0.844). In addition, 45.5% of patients with active cancer had a Rankin Scale score ≤3 at 3 months (65). Two recent studies involving large populations have shown no significant differences in the probability of discharge and cerebral hemorrhage after endovascular treatment in patients with stroke and cancer with metastasis versus those without cancer, although there were significant differences in in-hospital mortality (66, 67). Some studies have shown that endovascular treatment improves the quality of life in these patients (68–70).

Thus, decision-making must be a shared process between the patient and their family, preferably involving a team that includes a neurologist and oncologist, to accurately assess the risk–benefit balance of the different therapeutic measures (68).

Patients with stroke and cancer should be considered candidates for thrombolysis (rTPA or tenecteplase) and endovascular treatment.

After a stroke, the oncologist may have reservations about administering chemotherapy, concerned that the patient may be too weak to tolerate the possible side effects or that chemotherapy treatment could trigger another stroke (71). In this situation, a joint assessment by neurology and oncology is essential to assess treatment objectives, functional status, and overall risks and benefits.

## 7 Prognosis

The frequency of stroke recurrence in patients with CES-ONC ranges from 14 to 34%, and conventional ischemic stroke recurs in 15.7% of patients (72). D-dimer levels have traditionally been evaluated as a useful prognostic factor in these patients. Reductions in D-dimer levels after the initiation of antithrombotic treatment are associated with a better prognosis, including a lower risk of recurrence and improved survival (24).

Patients with CES-ONC have a poorer prognosis, long-term functional status, and survival than patients with cryptogenic stroke without cancer (71, 73).CES-ONC is associated with a poorer prognosis upon discharge and a tendency for longer stays in the Stroke Unit (74). In addition, the presence of both venous and arterial thromboembolisms was independently associated with poorer 1-year survival (58).

If patients present with NBTE, they have a significantly higher mortality rate of 80% and a stroke recurrence rate of 50% over a follow-up period of 6 months (70, 73).

However, in recent years, significant advances have been made in the field of cancer treatment. This has increased the survival and quality of life of many patients (74, 75). In the near future, genetic factors are expected to refine our prognosis. The driver gene KRAS aggravates cancer-associated stroke outcomes (76).

## 8 Discussion

To improve the management of these patients, we must maintain a high suspicion that the etiopathogenetic mechanisms described are present in patients in whom cancer and cryptogenic embolic ischemic stroke coexist.

The clinical presentation can be focal or multifocal, with elevated D-dimer levels. It is typical to find multifocal lesions in neuroimaging tests with the “three part sign.”

The management of acute stroke does not differ between patients with and without cancer; the presence of neoplastic disease should not be considered an absolute contraindication for treatment with intravenous thrombolysis or endovascular treatment.

These patients tend to have a poorer prognosis when NBTE occurs, often indicating the presence of tumor-induced platelet aggregation and metastasis (70).

The complexity of treatment arises from the fact that there are several possible mechanisms that determine the relationship between stroke and cancer; therefore, not all patients respond optimally to anticoagulation.

Low-molecular-weight heparin is usually used empirically when a hypercoagulable state is suspected, and the limited studies available support the use of direct oral anticoagulants as an option with similar efficacy. However, ongoing clinical trials are needed before evidence-based recommendations can be made.

On the other hand, in this condition, both platelets and the coagulation cascade are activated, and a two-way antithrombotic treatment strategy (a combination of antiplatelet agents and anticoagulants) could be a more comprehensive approach for this patient population (77, 78).

To accurately assess the risk–benefit balance, we must consider that this population also has an increased risk of hemorrhage.

For embolic cryptogenic ischemic stroke with occult neoplasia, the best screening strategy is unclear and we do not have adequate biomarkers. A current challenge in the field of cerebrovascular disease is to obtain a biomarker with high sensitivity and specificity for occult neoplasia in stroke. In this sense, the detection of microvesicles released by cancer cells with a specific RNA content has the characteristics of an ideal biomarker, as it is one of the main pathways of hypercoagulability that occurs in this condition and causes embolic stroke.

We must advance our ability to precisely define CES-ONC (79).

This includes identifying the presence of predictive factors for occult neoplasia, such as clinical factors (age, risk factors, and severity) and biomarkers (CRP, D-dimer), as well as neuroimaging findings (RNMC DWI: “three parts”) (80).

Regarding the impact of occult neoplasia on stroke, we can make an approximation based on data concerning its prevalence.

The percentage of patients with embolic stroke of undetermined origin (cryptogenic) was 13.5%, of which 2.8% had occult neoplasia.

The annual incidence of stroke is 187 per 100,000 inhabitants. The percentage of patients with occult neoplasia and embolic cryptogenic ischemic stroke among all strokes was 0.4%. Therefore, the incidence of stroke and occult neoplasia should be approximately three patients per year in a healthy area of 400,00 inhabitants.

A scale used to assess the risk of occult neoplasia in patients with cryptogenic embolic stroke is the OCCULT-5 score, which includes the following criteria: age ≥ 77 years, embolic stroke of undetermined source, multi-territorial infarcts, D-dimer levels ≥ 820 μg/L, and female sex. A score of ≥ 3 predicts occult neoplasia with a sensitivity of 64%, a specificity of 73%, a positive likelihood ratio of 2.35, and a negative likelihood ratio of 0.50 (81).

Having a biomarker with high sensitivity and specificity would enable the early diagnosis of cancer in this subgroup of patients, potentially improving survival outcomes due to the well-established benefits of early detection.

Stroke is a complication that must be considered in patients with cancer. Its diagnostic and therapeutic management have complexities that doctors who treat these patients must recognize to ensure appropriate and effective care.
